# Temperature Correction of Spectra to Improve Solute Concentration Monitoring by In Situ Ultraviolet and Mid-Infrared Spectrometries toward Isothermal Local Model Performance

**DOI:** 10.1021/acs.oprd.2c00238

**Published:** 2022-11-04

**Authors:** Magdalene W. S. Chong, Thomas McGlone, Ching Yee Chai, Naomi E. B. Briggs, Cameron J. Brown, Francesca Perciballi, Jaclyn Dunn, Andrew J. Parrott, Paul Dallin, John Andrews, Alison Nordon, Alastair J. Florence

**Affiliations:** † EPSRC Future Continuous Manufacturing and Advanced Crystallisation Research Hub, University of Strathclyde, 99 George Street, Glasgow G1 1RD, U.K.; ‡ WestCHEM, Department of Pure and Applied Chemistry, and Centre for Process Analytics and Control Technology (CPACT), University of Strathclyde, 295 Cathedral Street, Glasgow G1 1XL, U.K.; § EPSRC Centre for Innovative Manufacturing in Continuous Manufacturing and Crystallisation, Strathclyde Institute of Pharmacy and Biomedical Sciences, Technology and Innovation Centre, University of Strathclyde, 99 George Street, Glasgow G1 1RD, U.K.; ∥ Clairet Scientific, 17/18 Scirocco Close, Moulton Park Industrial Estate, Northampton NN3 6AP, U.K.

**Keywords:** temperature correction, process analytical technology, UV spectrometry, IR spectrometry, chemometrics

## Abstract

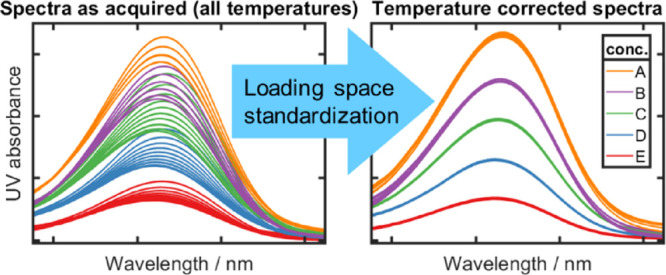

Changes in temperature can significantly affect spectroscopic-based methods for in situ monitoring of processes. As varying temperature is inherent to many processes, associated temperature effects on spectra are unavoidable, which can hinder solute concentration determination. Ultraviolet (UV) and mid-infrared (IR) data were acquired for l-ascorbic acid (LAA) in MeCN/H_2_O (80:20 w/w) at different concentrations and temperatures. For both techniques, global partial least squares (PLS) models for prediction of LAA concentration constructed without preprocessing of the spectra required a high number of latent variables to account for the effects of temperature on the spectra (root mean square error of cross validation (RMSECV) of 0.18 and 0.16 g/100 g solvent, for UV and IR datasets, respectively). The PLS models constructed on the first derivative spectra required fewer latent variables, yielding variable results in accuracy (RMSECV of 0.23 and 0.06 g/100 g solvent, respectively). Corresponding isothermal local models constructed indicated improved model performance that required fewer latent variables in the absence of temperature effects (RMSECV of 0.01 and 0.04 g/100 g solvent, respectively). Temperature correction of the spectral data via loading space standardization (LSS) enabled the construction of global models using the same number of latent variables as the corresponding local model, which exhibited comparable model performance (RMSECV of 0.06 and 0.04 g/100 g solvent, respectively). The additional chemometric effort required for LSS is justified if prediction of solute concentration is required for in situ monitoring and control of cooling crystallization with an accuracy and precision approaching that attainable using an isothermal local model. However, the model performance with minimal preprocessing may be sufficient, for example, in the early phase development of a cooling crystallization process, where high accuracy is not always required. UV and IR spectrometries were used to determine solubility diagrams for LAA in MeCN/H_2_O (80:20 w/w), which were found to be accurate compared to those obtained using the traditional techniques of transmittance and gravimetric measurement. For both UV and IR spectrometries, solubility values obtained from models with LSS temperature correction were in better agreement with those determined gravimetrically. In this first example of the application of LSS to UV spectra, significant improvement in the predicted solute concentration is achieved with the additional chemometric effort. There is no extra experimental burden associated with the use of LSS if a structured approach is employed to acquire calibration data that account for both temperature and concentration.

## Introduction

Accurate determination of solute concentration is critical to many industrial processes, including solubility determination,^1^ crystallization,^2^ and dissolution.^3^ Within the pharmaceutical industry, there has been increasing interest in quality by design approaches^4,5^ to tackle variability in processes. Key drivers for these methodologies are cost reduction and maximizing operational efficiency. Process analysis has been well established in the bulk chemical industry, and its introduction to pharmaceuticals and fine chemicals arose from the process analytical technology initiative, devised by the United States Food and Drug Administration as a system for designing, analyzing, and controlling manufacturing processes through measurements during processing.^6^ The incorporation of real time, in situ measurement capability is also crucial as off-line approaches will inevitably be susceptible to sampling artifacts and time delays, which may lead to inaccurate results and/or delayed process corrections. The development of attenuated total reflectance (ATR) probes that can be immersed in process media has allowed the routine deployment of mid-infrared (IR)^1^ and ultraviolet (UV)^7^ spectrometries for in situ solute concentration monitoring.^2,8−11^

For processes such as cooling crystallization,^2^ where operating over a temperature range is inherent to the process, strategies to mitigate temperature effects, such as band shifting and broadening,^12^ on spectra must be explored for successful monitoring and control. Accounting for the temperature sensitivity of inter- and intramolecular interactions^13−15^ during in situ measurements has been achieved using some relatively simple approaches. For example, a calibration procedure has been reported that directly models the effects of temperature on IR absorbance.^16^ Nine samples of different concentration/temperature combinations were required to determine the temperature influence on a selected solute peak height with respect to its concentration in solution. A similar procedure has been employed for UV data where temperature dependence was remedied by using the first derivative absorbance spectra at two single wavelengths and applying either a linear^17^ or a nonlinear^18^ calibration function.

Multivariate calibration procedures^19^ may also be adopted to account for temperature effects on spectra. For solute concentration measurement by IR spectrometry of L-glutamic acid (LGA) in aqueous solution,^20^ a partial least squares (PLS) model with five latent variables was used. However, while it was noted that the observed variation in LGA absorbance with temperature was small, a relatively high number of latent variables was required. The importance of sample selection for construction of PLS models has been studied for the monitoring of LGA concentration in water by IR spectrometry, by comparison of models constructed using samples of concentrations and temperatures from different regions of the phase diagram.^21^ Two of the models were constructed by combining samples from the metastable region with either all of the undersaturated samples or a selection of undersaturated samples just below the solubility line. However, the models constructed solely from the samples in the metastable or undersaturated zones demonstrated better overall predictive performance during those respective regimes within a cooling crystallization process.

Simple multivariate approaches are sometimes insufficient to account for the effects of temperature upon spectra.^22^ A number of more advanced multivariate methodologies has been applied for the correction of temperature fluctuations,^23,24^ principally for near-infrared measurements.^25−29^ Methods that rely on temperature independence^30^ are rare, and many that have been developed to simulate temperature independence are difficult to implement owing to the substantial modeling input and expertise required.^27,31−35^ Standardization approaches, including piecewise direct standardization^36,37^ and loading space standardization (LSS),^12,38^ have been reported to correct for complex nonlinear spectral variations arising from changes in temperature. With fewer parameters to determine, LSS is comparatively more straightforward to implement^12,39^ and the outcome of standardizing all spectra to be as though they were measured at the same temperature is conceptually reasonable. Details of the underlying mathematical framework for LSS are described elsewhere.^12^ Briefly, the nonlinear effects of temperature upon spectral absorbance are modeled using a second-order polynomial. Using singular value decomposition, the spectral data matrix can be expressed in terms of scores and loadings. The effect of temperature in the loadings is then modeled by fitting a second-order polynomial. Upon deciding the number of factors (components) required, a loading matrix is calculated for the required temperature, which can then be used to standardize (transform) the spectrum of a sample measured at a specific temperature to the required temperature.

Although LSS has been applied to IR data to remove the effects of temperature, especially where common preprocessing methods such as standard normal variate (SNV) and multiplicative scattering correction (MSC) were less effective,^42^ its application to UV data has not been reported. Variations in temperature result in changes to the peak position, width, and/or absorbance of UV spectra owing to changes in the electronic environment from solute–solvent interactions,^40,41^ which has been observed with in-line UV measurements of solute concentration during crystallization processes.^17,18^ For ATR measurements, temperature sensitivity can also be ascribed to changes in solution density and path length (penetration depth of the evanescent wave changes owing to temperature-induced changes in the refractive index of the liquid medium),^3^ while changes in the refractive index for the sapphire crystal are neligible.^7^ Attempted treatment of these temperature-dependent responses via SNV and MSC, for UV spectra to monitor solute concentration during dissolution, did not afford significant improvement.^3^ LSS may be more effective, as demonstrated for IR data.^42^

**Chart 1:**
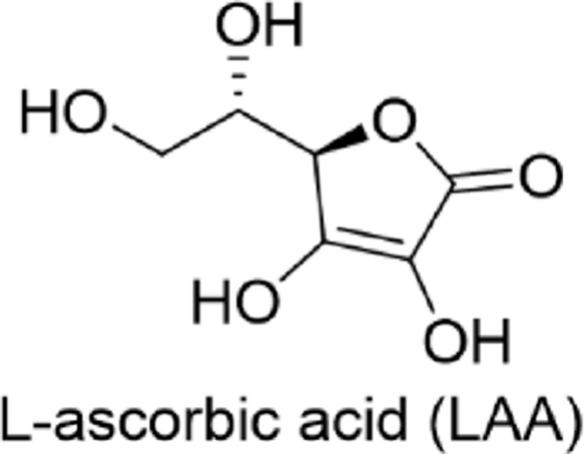
Chemical Structure of l-Ascorbic Acid (LAA)

Here, we report the application of UV and IR spectrometries for the determination of solubility diagrams for a model compound l-ascorbic acid (LAA, Chart 1) in an MeCN/H_2_O (80:20 w/w) solvent mixture. LAA was selected as a relatively inexpensive, commercially available compound with no reported polymorphic behavior. An MeCN/H_2_O (80:20 w/w) solvent mixture was chosen in the interest of working with a reasonable solid loading at desupersaturation. PLS calibration models were constructed, using no or minimal preprocessing, for prediction of LAA concentration from UV or IR spectra. Isothermal local models were also constructed to set a “benchmark” performance in the absence of temperature effects. Temperature correction of the spectra by LSS was explored and improvements in model performance compared to the local models. This is the first reported example of LSS being used for removal of temperature effects from UV spectra. The solubility diagrams obtained by UV and IR spectrometries were compared to those determined using the more traditional techniques of transmittance and a gravimetric method.

## Experimental Section

### Materials and Methods

LAA (reagent grade, Sigma-Aldrich) and MeCN (HPLC grade, Fisher Scientific) were used as received without further purification. Deionized water from an in-house Milli-Q (Millipore) purification system was used for all experiments.

**Figure 1:**
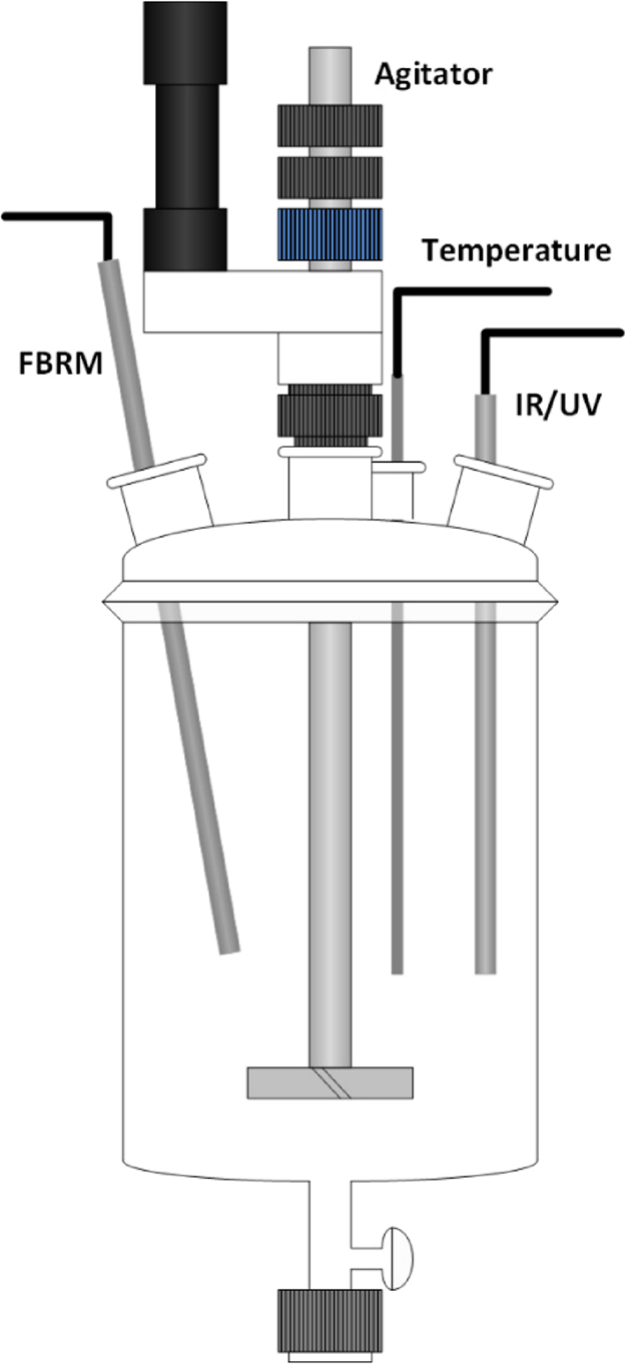
Schematic of the experimental setup. The vessel shown was used as part of the OptiMax platform including temperature, focused beam reflectance measurement (FBRM), and IR or UV probes.

Small-scale experiments were performed in a Crystalline (Technobis Crystallization Systems) platform for determination of solubility. The platform was operated with the Crystalline (version 2.15, Technobis Crystallization Systems) software and all analysis was performed using CrystalClear (version 1.0, Technobis Crystallization Systems). Larger scale experiments were performed in an OptiMax 1001 (Mettler Toledo) workstation of 1 L capacity (Figure 1), equipped with an in-line hastelloy PT100 temperature sensor. The system was operated using iControl (version 5.2, Mettler Toledo) software, which included temperature control. Evaporation of solvent at high temperatures was avoided by the use of a reflux condenser. For IR experiments, a ReactIR 15 (Mettler Toledo) unit was added to the workstation with iC IR (version 4.3, Mettler Toledo) incorporated into the iControl software. A fiber coupled diamond ATR probe was immersed in the solution. The IR spectra were collected with a resolution of 8 cm^–1^ with an air background used as the reference. For UV experiments, an MCS561 (Carl Zeiss) spectrophotometer was fiber coupled to a 6 mm diameter 3-bounce sapphire ATR probe (Hellma). The probe was immersed in the solution and the UV instrument was operated using ASPECT Plus (version 1.76, Zeiss) software, which operated independently of iControl. The UV spectra were acquired using an integration time of 40.9 ms with subtraction of the dark current and an MeCN/H_2_O (80:20 w/w) solvent background was used as the reference. For all larger scale experiments, a ParticleTrack G400 (Mettler Toledo) was used with iC FBRM (4.3, Mettler Toledo) incorporated in the iControl software, and for the solubility experiments, a particle vision microscope (PVM) V819 (Mettler Toledo) probe with online image acquisition software (version 8.3, Mettler Toledo) was employed.

### Calibration and Solubility Experiments Using IR and UV Spectrometries

For the calibration experiments, a fixed concentration of LAA in an MeCN/H_2_O (80:20 w/w) solvent mixture was prepared at ca. 70 °C and transferred as a clear mixture to the OptiMax workstation. A stepped cooling profile was completed over the range of 75 to −10 °C. Five LAA concentrations (4, 8, 12, 16, and 20 g/100 g solvent) were included in the calibration set, and two validation experiments (5 g/100 g solvent at 20 °C and 15 g/100 g solvent at 65 °C) were performed for each technique. These experiments were held at a constant temperature for a period of ca. 30 to 70 min. For the solubility experiments, an LAA-solvent slurry (30 g/100 g solvent) was prepared and a stepped heating profile (5 °C min^–1^ ramps and 30 min hold periods) from 0 to 70 °C at increments of 10 °C was completed with an excess of solid present throughout to ensure a saturated supernatant.

### Solubility Experiments by Transmittance or Imaging

The Crystalline platform consists of eight glass reactors, with a working volume of ca. 6 mL, which are stirred by overhead agitators. Transmission of light through the solution was recorded to identify clear points for solubility and an in-built particle visualization module allowed for in situ imaging measurements with 2.8 μm/pixel resolution. Twelve slurries of LAA in MeCN/H_2_O (80:20 w/w) in the concentration range 6 to 20 g/100 g solvent were prepared and stored overnight at −20 °C prior to measurement of solubility to minimize initial dissolution. Heating ramps of 0.1 °C min^–1^ were applied and the clear points identified.

### Gravimetric Solubility Experiments

Solubility experiments were performed in the Crystalline platform to achieve stable temperatures. Ten slurries of LAA in MeCN/H_2_O (80:20 w/w) in the concentration range 6 to 25 g/100 g solvent were prepared and held at a constant temperature for a period of 4 h. The experiments were designed such that an excess of solid was always present. The slurries were then immediately filtered with prewarmed syringes and filters into preweighed vials. The clear mixtures were evaporated to dryness allowing the solid mass to be determined. The solubility measurement was repeated four times for each concentration.

### Data Analysis

The spectra corresponding to each sample (concentration/temperature combination) were assigned using the Multifile tool in Grams (version 9, Thermo) software. The data were cut to only include the stabilized regions, i.e., sections where the temperature was stabilized and the spectral signal had plateaued, resulting in a different number of replicates per sample (Table S1).

Subsequent data analysis was performed in Matlab (version R2018a, Mathworks) using PLS_Toolbox (version 8.6.2, Eigenvector Research) for the construction of PLS models. A random subset cross validation procedure (3 splits, 20 iterations) was employed, and the minimal number of latent variables was selected which provided a suitable accuracy according to the root mean square error of cross validation (RMSECV). First derivative IR and UV spectra were obtained by application of a Savitzky–Golay filter to the full spectra, calculated using 7-point and 15-point filter widths, respectively, and a second-order polynomial. All PLS models for the IR and UV data were constructed using the spectral ranges 1831 to 709 cm^–1^ and 218 to 285 nm, respectively, with mean centering of the spectra.

Construction of LSS models was performed in Matlab, and for all LSS models, two LSS components were selected as determined from the singular value decomposition results. The LSS models were constructed using the samples at the three lower LAA concentrations (4, 8, and 12 g/100 g solvent) where spectra were acquired at all 10 temperatures in the design space (Table 1). The number of spectra per sample (individual concentration and temperature combination) used for the construction of the LSS models was determined by the minimum number of spectra available for a sample in each dataset, which are 14 and 22 for the IR and UV datasets, respectively (Table S1). The LSS models were constructed using the aforementioned spectral ranges used for construction of the PLS models. The LSS models were applied to the full calibration datasets to transform the spectra so that they appear as though they were all acquired at 50 °C.

**Table 1:** Concentrations and Temperatures of the Samples in the Calibration Sets^a^

[LAA]/g/100 g MeCN/H_2_O (80:20 w/w)	temperatures in the calibration set/°C									
4	–10*	0*	10*	20*	30*	40*	50*	60*	70*	75*
8	–10*	0*	10*	20*	30*	40*	50*	60*	70*	75*
12	–10*	0*	10*	20*	30*	40*	50*	60*	70*	75*
16					30	40	50	60	70	75
20							50	60	70	75

a Samples used for construction of the LSS model are indicated by asterisks.

## Results

### Calibration Procedure for Monitoring the Concentration of LAA by IR and UV

An initial approximation of the solubility diagram, as a function of temperature, was obtained at the small scale using a combination of transmittance and imaging to identify clear points at various concentrations. The design space for the calibration model was subsequently specified in terms of temperature (−10 to 75 °C) and concentration (4 to 20 g/100 g solvent) of LAA in MeCN/H_2_O (80:20 w/w). Five concentrations were selected and separate variable temperature experiments performed for each concentration using the 1 L crystallizer (Figure 1). Each experiment commenced with a clear mixture, and completion of the experiment was determined by FBRM monitoring; upon nucleation, the concentration of the supernatant becomes unknown. A stepped cooling profile was employed to allow sufficient equilibration time for the crystallizer contents and to acquire IR or UV spectra of sufficiently good signal to noise at a well-defined temperature. The calibration set consisted of 40 samples (Table 1); spectra acquired when either nucleation had occurred or the temperature was not stabilized were removed.

**Figure 2:**
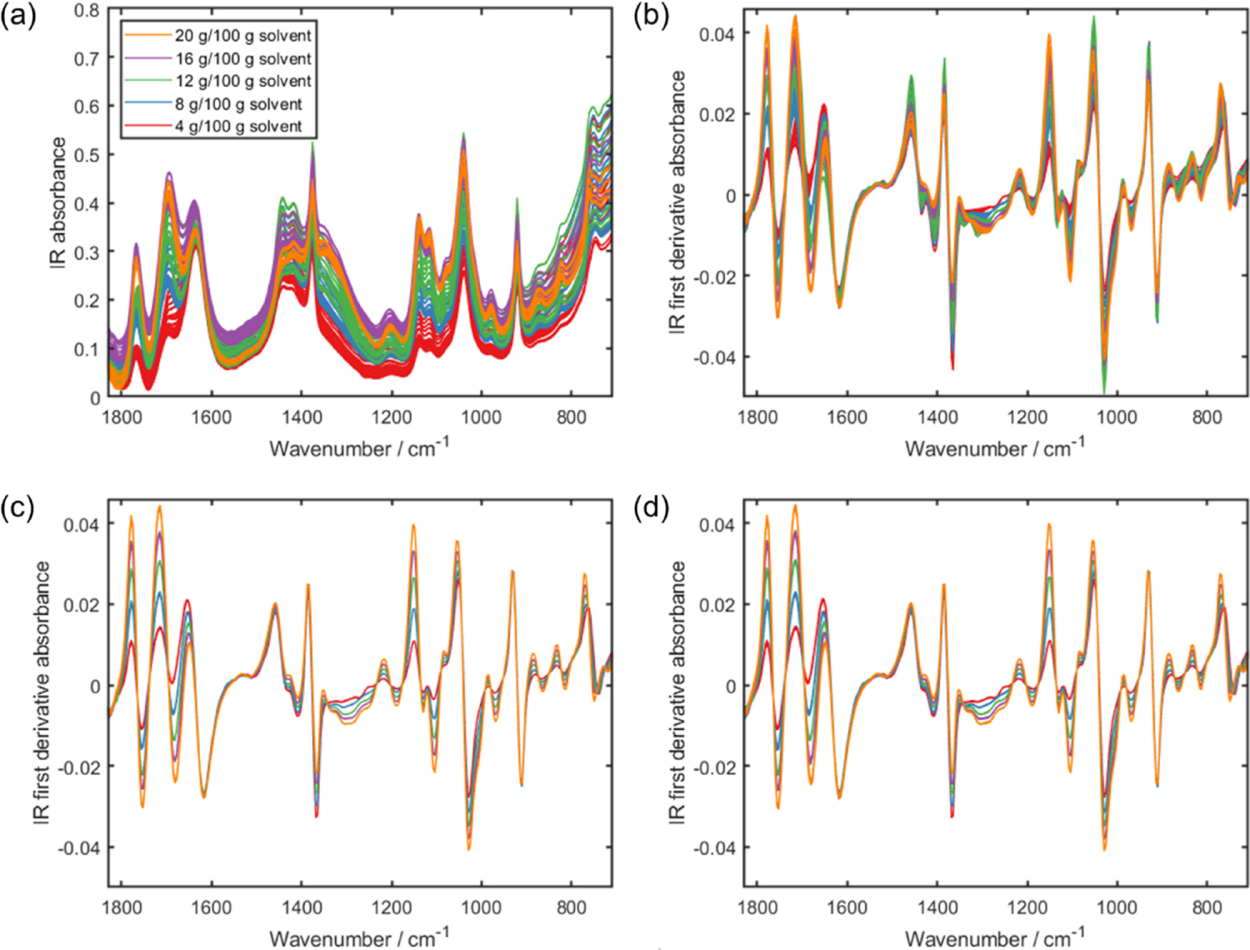
Overlay of IR calibration spectra acquired for different concentrations of LAA in MeCN/H_2_O (80:20 w/w): (a) all absorbance spectra, (b) all first derivative spectra, (c) first derivative spectra acquired at 50 °C, and (d) first derivative spectra transformed via LSS so they are as though they were acquired at 50 °C.

**Figure 3:**
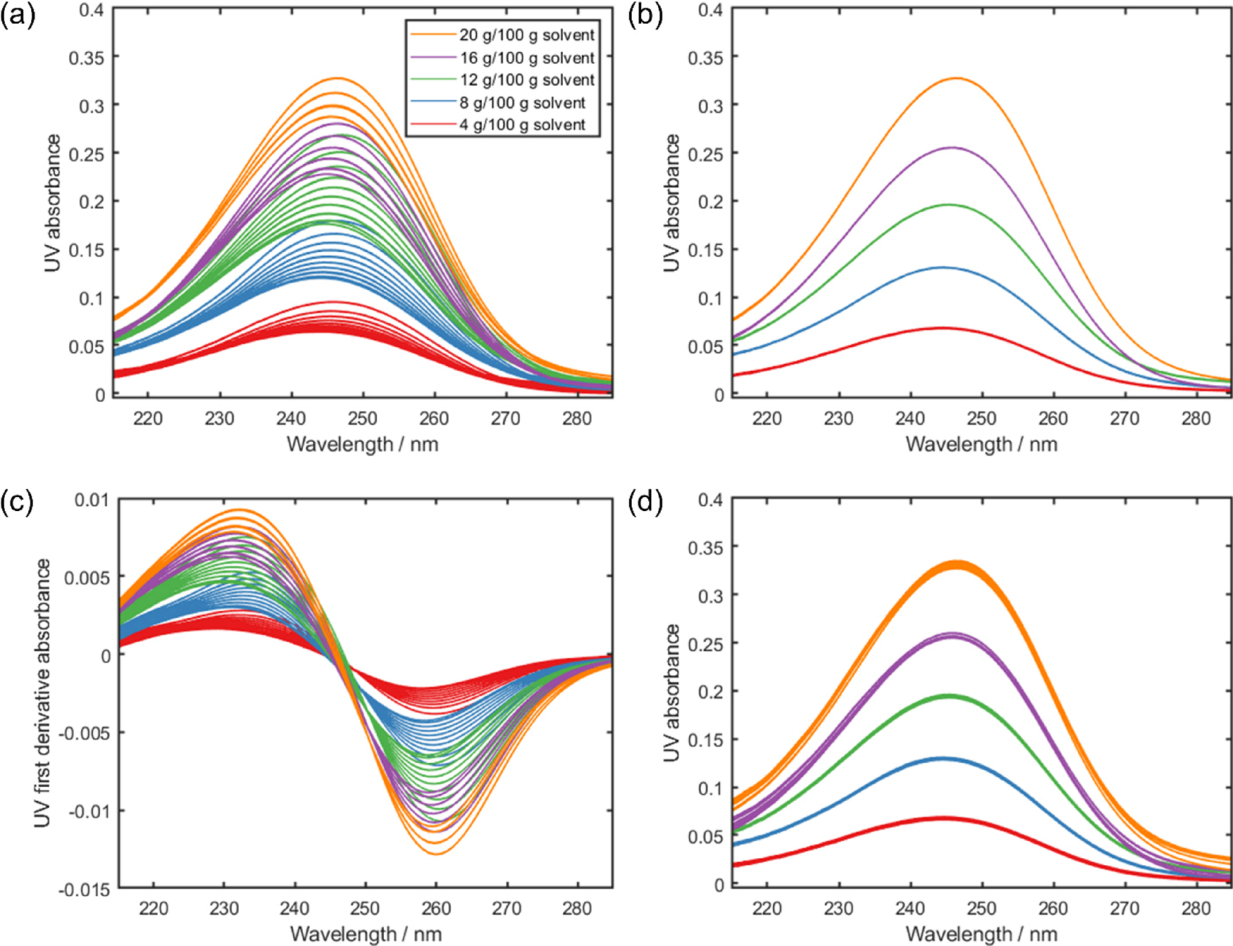
Overlay of UV calibration spectra acquired for different concentrations of LAA in MeCN/H_2_O (80:20 w/w): (a) all absorbance spectra, (b) absorbance spectra acquired at 50 °C, (c) all first derivative spectra, and (d) all absorbance spectra after transformation via LSS so they are as though they were acquired at 50 °C.

The IR spectra are complex and information rich with many molecular vibrational features (Figure 2a). Some of these features vary considerably with temperature, for example, the ν(C=O) and ν(C=C) modes of the LAA solute at ca. 1765 and 1695 cm^–1^, respectively. The solvent bands, for example, δ(CH) and ν(CC) observed for MeCN at ca. 1375 and 920 cm^–1^, respectively, vary to a much lesser extent with temperature. In contrast, the UV spectra feature a single peak (Figure 3a), where the absorbance is affected by both concentration (denoted by the different colored lines) and temperature (represented by the multiple lines for a given concentration). While the effect of temperature is evident in the absorbance spectra, its effect is more apparent when focusing on specific IR and UV solute peaks over time (Figures S1 and S2, respectively).

The effects of temperature can also be visually compared with the spectra acquired at a single temperature. The lowest temperature that spectra were acquired at for all concentrations is 50 °C (Table 1). The UV spectra acquired at 50 °C feature five distinct bands (Figure 3b), which is expected as concentration should be the only factor affecting the absorbance of isothermal samples. Owing to the baseline effects observed in the IR absorbance spectra (Figure 2a), the temperature effects are better visualized in the first derivative spectra. Multiple bands are once again observed in the first derivative IR spectra (Figure 2b); the 50 °C isothermal subsets of these are five distinct bands (Figure 2c). The features in the IR first derivative spectra corresponding to the solute and solvent further highlight the effect of temperature on the solute. As the solvent is consistent across all spectra, the solvent peaks are overlaid in the isothermal spectra (Figure 2c). This can be observed for the solvent features at ca. 1620, 1380, and 920 cm^–1^ corresponding to δ(OH), δ(CH), and ν(CC) vibrational modes, respectively. The solute peaks, which exhibit a range of absorbances in the first derivative spectra (Figure 2b), are resolved into distinct bands for each concentration in the 50 °C spectra (Figure 2c). While the ν(C=O) and ν(C=C) features at ca. 1765 and 1695 cm^–1^, respectively, are grouped by concentration in the first derivative spectra (Figure 2b), the bands are more distinct in the isothermal spectra (Figure 2c). The ester ν(CO) vibration at ca. 1200 cm^–1^ and multiple alcohol δ(OH) and ν(CO) coupled vibrational modes observed at ca. 1140, 1115, and 1040 cm^–1^ are also similarly resolved in the isothermal spectra (Figure 2c).

The range 1831 to 709 cm^–1^ was selected for construction of PLS models from the IR data as it includes solute absorbance peaks with a reliable spectroscopic signal and excludes regions of high noise owing to absorbance of the diamond ATR element. Global PLS models were constructed on the mean centered absorbance and first derivative spectra (Table 2). An improved calibration model performance is achieved with application of first derivative preprocessing, with the RMSECV more than halved. A high number of latent variables is required to account for the temperature effects on the spectra. For an indication of the “benchmark” model performance where the effects of temperature have been removed, an isothermal local model may be constructed from the first derivative spectra acquired at 50 °C (Figure 2c). Compared to the global models, the local model constructed from the first derivative spectra uses fewer latent variables with improvements in the calibration model performance (Table 2).

**Table 2:** Performance Metrics of the PLS Models for the Prediction of LAA Concentration by IR and UV^a^

	data and preprocessing	LVs	calibration		cross validation		validation (5 g/100 g solvent at 20 °C)					validation (15 g/100 g solvent at 65 °C)				
			RMSE /g/100 g solvent	*R*^2^	RMSE /g/100 g solvent	*R*^2^	RMSE	mean	min	max	RSD	RMSE	mean	min	max	RSD
							/g/100 g solvent				/%	/g/100 g solvent				/%
IR	global	4	0.1540	0.998935	0.1554	0.998923	0.4912	5.49	5.37	5.55	0.88	0.2400	15.24	15.21	15.27	0.10
	global, 1st derivative	5	0.0611	0.999833	0.0621	0.999829	0.2098	4.79	4.73	4.85	0.52	0.0767	15.07	15.02	15.13	0.19
	global, 1st derivative, LSS to 50 °C	3	0.0394	0.999930	0.0399	0.999929	0.2098	4.79	4.75	4.85	0.42	0.0229	14.99	14.95	15.04	0.14
	local (50 °C), 1st derivative	3	0.0343	0.999959	0.0367	0.999954										
UV	global	6	0.1811	0.998678	0.1819	0.998668	0.3238	5.32	5.19	5.46	0.92	0.1673	14.85	14.64	15.02	0.52
	global, 1st derivative	3	0.2243	0.997972	0.2253	0.997959	0.1051	5.08	4.91	5.22	1.22	0.3061	14.70	14.51	14.89	0.52
	global, LSS to 50 °C	2	0.0621	0.999845	0.0622	0.999844	0.0096	5.01	4.99	5.02	0.09	0.1778	14.82	14.79	14.84	0.07
	local (50 °C)	2	0.0142	0.999994	0.0145	0.999993										

a LVs = latent variables; RMSE = root mean square error; *R*^2^ = correlation coefficient; RSD = relative standard deviation.

Similarly, PLS calibration models were also constructed for the prediction of LAA concentration by UV spectrometry. The range 215 to 285 nm was used, which includes the LAA absorbance. Once again, a high number of latent variables (Table 2) is required for the global models constructed on the mean centered absorbance (Figure 3a) and first derivative (Figure 3c) spectra. An isothermal local model was constructed using the spectra acquired at 50 °C (Figure 3b) for an indication of the model performance without any effects of temperature. There is a significant improvement in the local model performance, with fewer latent variables required and the RMSECV reduced by an order of magnitude (Table 2). Comparing the global models, larger errors are obtained from the models constructed using the UV data than for the IR data (Table 2), suggesting a greater temperature sensitivity for the UV data. The local models indicate that the RMSECV for the model constructed using the UV data is more than half that when using the IR data (Table 2).

To construct LSS models for temperature correction of spectra, a dataset is required comprising multiple samples (concentrations) measured at a number of temperatures.^12^ A limitation that commonly occurs for cooling crystallization data is that the LSS model input cannot include the full range of the design space (owing to solubility limits).^39,42^ For this dataset, the LSS models were constructed using the samples at the three lower LAA concentrations (4, 8, and 12 g/100 g solvent) where spectra were acquired at all 10 temperatures in the design space (Table 1). While detailed exploration of the construction and application of LSS models is outside the scope of this paper, the strategy elected here is to include all temperatures in the LSS model input and to use the LSS model to transform the spectra to 50 °C, since that is the lowest temperature at which spectra were acquired for all of the concentrations. For the UV data, the LSS model with two components was constructed and then applied to the entire calibration dataset to transform the spectra so that they appear as though they were all acquired at 50 °C (Figure 3d). A similar procedure was applied to the IR data (Figure 2d); however, the LSS model was constructed using the IR spectra after application of first derivative preprocessing (Figure 2b).

Upon applying LSS, the multiple absorbance bands observed for each concentration in the UV spectra (Figure 3a) become overlaid and are grouped in five distinct bands (Figure 3d). The spectra transformed to 50 °C by LSS (Figure 3d) visually resemble those acquired at 50 °C (Figure 3b). This suggests that LSS is appropriate for removal of temperature effects from UV spectra, with this being the first application of LSS to UV data to our knowledge. The IR spectra transformed to 50 °C by LSS (Figure 2d) also visually resemble those that were acquired at 50 °C (Figure 2c).

The PLS calibration models constructed from the temperature-corrected spectra (Figures 2d and 3d), via application of LSS, exhibited improved calibration performance (Table 2). For the model constructed from the LSS-corrected IR spectra, the improved RMSECV and correlation coefficient (*R*^2^) show that a global model can be constructed with similar performance to the local model. A significant improvement in calibration model performance is achieved with the model constructed using the LSS-corrected UV spectra, particularly the threefold reduction in RMSECV. Additionally, with LSS correction of the data, the number of latent variables required for the subsequent PLS models was reduced to the same as for the local models. In particular, LSS is successful at removing the effects of temperature from the UV data compared to the other preprocessing methods studied (Table 2).

The spectra from the validation experiments (5 g/100 g solvent at 20 °C and 15 g/100 g solvent at 65 °C) were used to compare the performance of the PLS models with and without temperature correction by LSS (Table 2). The validation samples were selected to be at relatively low and high concentrations and temperatures within the design space. Slight improvements in the accuracy and precision are obtained with the predicted values for the IR model with temperature correction via LSS compared to the global models constructed without LSS correction. For the models constructed using the UV data, temperature correction of the spectra via LSS results in improved accuracy. The improvement in the relative standard deviation (RSD) by at least a factor of 7 (Table 2) highlights the enhanced precision.

### Determining the Solubility of LAA by IR and UV Spectrometries

**Figure 4:**
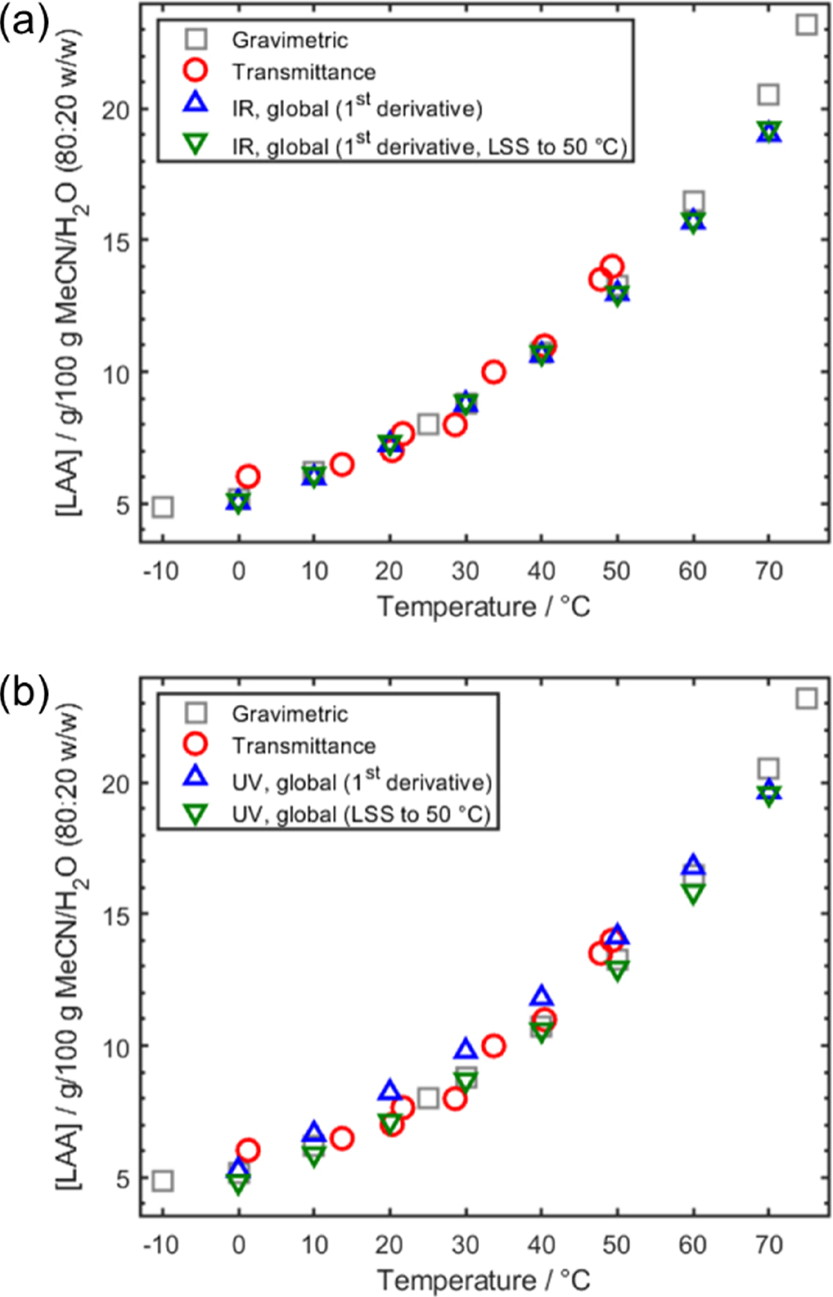
Variable temperature solubility diagrams for LAA in MeCN/H_2_O (80:20 w/w) obtained using gravimetric, transmittance, and (a) IR and (b) UV methods.

To obtain IR (Figure S3) and UV (Figure S4) data for determining the solubility of LAA in an MeCN/H_2_O (80:20 w/w) solvent system, spectra were acquired of a slurry of LAA during a stepped heating profile. The amount of LAA to suspend was chosen to ensure a constant presence of excess solids, which was verified by in situ FBRM and PVM monitoring. Confirmation that the thermodynamic solubility was reached during the hold period can be observed by the plateauing of the spectroscopic signals (Figures S3 and S4). To determine the solubility at each temperature, a predicted LAA concentration was obtained for the final spectrum at that holding temperature using the best global PLS model without LSS temperature correction and the PLS model constructed using the LSS-corrected spectra previously described (Table 2). The final spectrum from each step was selected as this should be the point at which equilibrium should have been reached, and the contents of the slurry are at the thermodynamic solubility. This enabled solubility curves to be determined from the IR and UV data. These solubility values were compared to those obtained in the initial assessment performed in the Crystalline platform and those determined from a conventional gravimetric approach^2^ (Figure 4 and Table S2).

The solubilities obtained from the monitoring of heated slurries via IR (Figure 4a) and UV (Figure 4b) spectrometries exhibit excellent agreement with those obtained by the more traditional methods of solubility determination via transmittance/imaging (Crystalline) and gravimetric means. For both IR and UV methods, the solubility values obtained appear to be in better agreement with the gravimetrically determined values. This is perhaps not overly surprising as the temperature measurement in the Crystalline platform is indirect and inferred from an internal calibration. The gravimetric approach is the most fundamental and direct technique, which would be expected to be the most accurate. Temperature correction by LSS aligns the predicted solubilities toward those that have been determined gravimetrically. For the UV models, the mean bias values with and without application of LSS are −0.39 and 0.42 g/100 g solvent, respectively (Figures S5 and S6). However, application of LSS significantly reduces the bias range from 1.94 to 0.85 g/100 g solvent. Similarly, there are also improvements in the solubility predictions by IR spectrometry with application of LSS. The mean and range of the bias are reduced from −0.43 to −0.34 and 1.49 to 1.35 g/100 g solvent, respectively (Figures S5 and S6). Comparing the two techniques, the mean biases for the models with application of LSS are comparable, with a slightly better performance using IR spectrometry (Figure S6). However, the bias range is considerably smaller for UV spectrometry, which is consistent with the validation results (Table 2).

## Discussion

Temperature effects are evident in the IR and UV spectra of LAA in MeCN/H_2_O (80:20 w/w), observed visually as multiple lines in the absorbance spectra (Figures 2a and 3a). The effects of temperature on spectra are particularly apparent upon comparison with spectra acquired at a single temperature (Figures 2c and 3b). As the IR spectra feature vibrational modes from both the solute and solvent, temperature effects are especially evident on the solute peaks. Expanding the approach on visual comparison of the spectra, global and isothermal local PLS models were constructed from the datasets for prediction of LAA concentration. While using isothermal local models is not a practical approach to applying models to a process, it provides an indication of the “benchmark” model performance by mimicking a scenario in which temperature effects have been removed. For both datasets, the local models required fewer latent variables and exhibited superior calibration model performance (Table 2). For both IR and UV datasets, using a high number of latent variables appears to be a relatively straightforward method of accounting for temperature effects in the global models. Increasing the number of latent variables accounts for nonlinearities in the data; however, this approach also incurs a risk of overfitting. The inferior performance of the global models compared to the local models suggests that significant improvements may be achieved with removal of temperature effects from the spectra.

Using temperature correction via LSS, spectra are transformed so they appear as though they were acquired at 50 °C (Figures 2d and 3d) and visually resemble the corresponding isothermal spectra (Figures 2c and 3b). The PLS calibration models constructed from the temperature-corrected spectra exhibit improved calibration model performance, approaching that of the respective local models (Table 2). Although it is not expected that the LSS correction will replicate the PLS model performance of the analogous local model, substantial improvements have been achieved using LSS temperature correction compared to simpler strategies of selecting a high number of latent variables or applying a first derivative. The significant improvement in the global model performance toward the local model performance demonstrates that LSS can facilitate a strategy for global model construction that approximates an impractical isothermal approach.

The improvements in calibration model performance upon removal of temperature effects are also demonstrated in the validation results. In particular, LSS temperature correction significantly improves the precision of the UV model. This was expected as the effect of temperature was more pronounced for the UV spectra (Figure 3), compared to the IR spectra (Figure 2), with the local model suggesting that substantial model improvement could be attained with successful removal of temperature effects (Table 2).

The models were applied for determination of solubility by IR and UV spectrometries. While relatively accurate solubilities can be determined by IR and UV using calibrations constructed with minimal preprocessing, these can be further improved with the application of temperature correction by LSS. This is shown by the solubilities predicted from the models with removal of temperature effects by LSS being in better agreement with the gravimetrically determined values. The balance between prediction accuracy and chemometric effort required depends on the purpose of the measurement.

## Conclusions

In this study, global PLS models were constructed using three preprocessing methods: none, first derivative, and LSS. Isothermal local models were also constructed from subsets of the calibration datasets to assess the potential gains in calibration model performance with removal of temperature effects from the spectra. Inclusion of a high number of latent variables is a relatively simple approach to account for temperature effects, with the minimal chemometric effort likely to be more appropriate for early phase development. However, the global models constructed from datasets corrected via LSS exhibit significantly improved model performance toward that of the impractical isothermal local models. For real-time monitoring and control, the improved accuracy provided by LSS is likely needed, and thus, expending the additional chemometric effort is worthwhile. Experimentally, the data acquisition would adopt a structure that accounts for both concentration and temperature. Whether the chemometric expertise for deploying an advanced algorithm is necessary depends on the accuracy and precision required of the calibration model.

## Supporting Information Available

Number of spectra per sample (concentration–temperature combination), absorbance trends, LAA gravimetric solubility data, and bias of UV and IR solubility predictions (PDF)

## Notes

All data underpinning this publication are openly available from the University of Strathclyde KnowledgeBase at https://doi.org/10.15129/01321c72-321f-4f55-8541-7f0644a6081f.
