# Supplementary material for: Temperature Correction of Spectra to Improve Solute Concentration Monitoring by In Situ Ultraviolet and Mid-Infrared Spectrometries toward Isothermal Local Model Performance
Source: Org Process Res Dev. 2022 Nov 4;26(11):3096–105. doi: 10.1021/acs.oprd.2c00238 (PMC9680019; doi:10.1021/acs.oprd.2c00238)
Supplement: Supplementary file 1 — op2c00238_si_001.pdf [file op2c00238_si_001.pdf]

Supporting information: **Temperature correction of spectra to improve solute concentration monitoring by *in situ* ultraviolet and mid-infrared spectrometries towards isothermal local model performance**

Magdalene W. S. Chong[ab§], Thomas McGlone[a§], Ching Yee Chai[c], Naomi E. B. Briggs[c], Cameron J. Brown[a], Francesca Perciballi[c], Jaclyn Dunn[bc], Andrew J. Parrott[b], Paul Dallin[d], John Andrews[d], Alison Nordon[ab]* and Alastair J. Florence[a]*

[a] EPSRC Future Continuous Manufacturing and Advanced Crystallisation Research Hub, University of Strathclyde, 99 George Street, Glasgow, G1 1RD, United Kingdom

[b] WestCHEM, Department of Pure and Applied Chemistry and Centre for Process Analytics and Control Technology (CPACT), University of Strathclyde, 295 Cathedral Street, Glasgow, G1 1XL, United Kingdom

[c] EPSRC Centre for Innovative Manufacturing in Continuous Manufacturing and Crystallisation, Strathclyde Institute of Pharmacy and Biomedical Sciences, Technology and Innovation Centre, University of Strathclyde, 99 George Street, Glasgow, G1 1RD, United Kingdom

[d] Clairet Scientific, 17/18 Scirocco Close, Moulton Park Industrial Estate, Northampton, NN3 6AP, United Kingdom

Table S1 Number of spectra (UV/IR) per calibration concentration/temperature combination.

[LAA] / g/100 g solvent	Temperature / °C									
(MeCN/H_2O (80:20 w/w))	−10	0	10	20	30	40	50	60	70	75
4	78/15	64/15	79/15	71/18	64/15	72/15	79/18	78/20	93/20	29/15
8	86/29	72/30	79/31	78/36	85/30	86/30	86/36	85/40	93/40	29/30
12	86/16	79/15	65/15	78/18	86/16	86/15	86/18	85/20	93/21	22/15
16	-	-	-	-	93/14	93/14	85/18	85/20	93/20	36/14
20	-	-	-	-	-	-	86/18	85/19	64/20	22/14

LAA = L-ascorbic acid

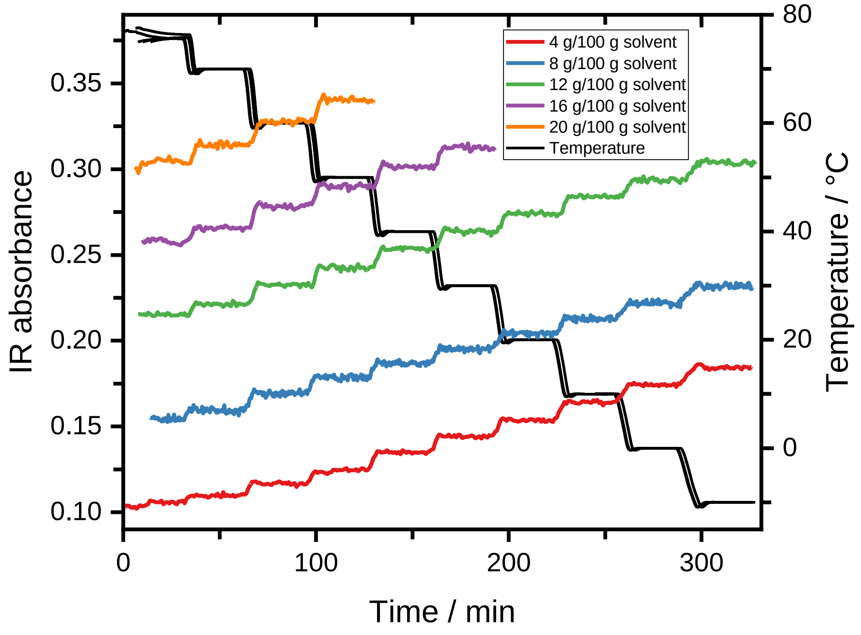

Figure S1. IR absorbance of the carbonyl band over the spectral range 1732 to 1678 cm^{-1}, calculated with a single point baseline correction, for the five variable temperature experiments to obtain the IR calibration data for LAA in MeCN/H_2O (80:20 w/w). Spectra that were collected where nucleation had occurred (monitored by FBRM) were removed from the dataset.

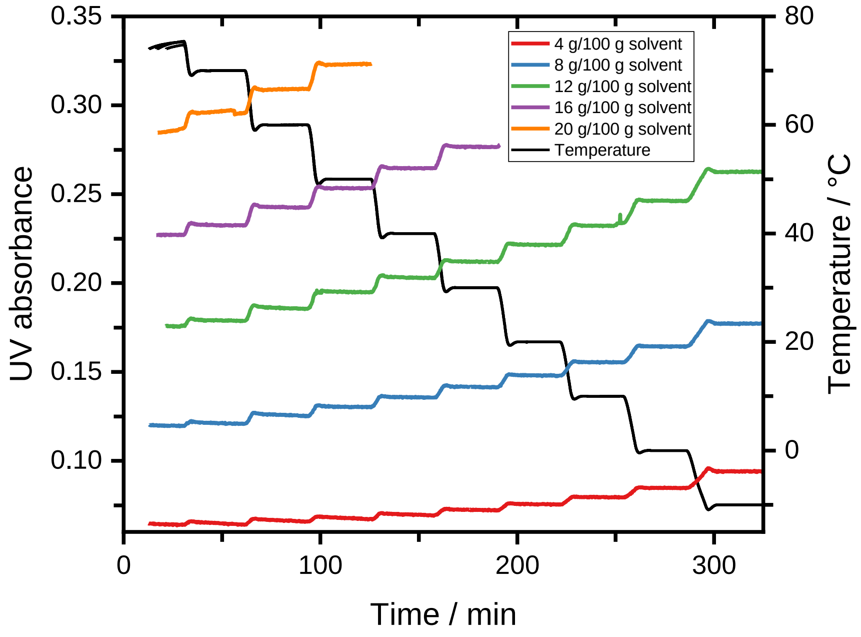

Figure S2. UV absorbance of LAA at 244 nm for the five variable temperature experiments to obtain the UV calibration data for LAA in MeCN/H_2O (80:20 w/w). Spectra that were collected where nucleation had occurred (monitored by FBRM) were removed from the dataset.

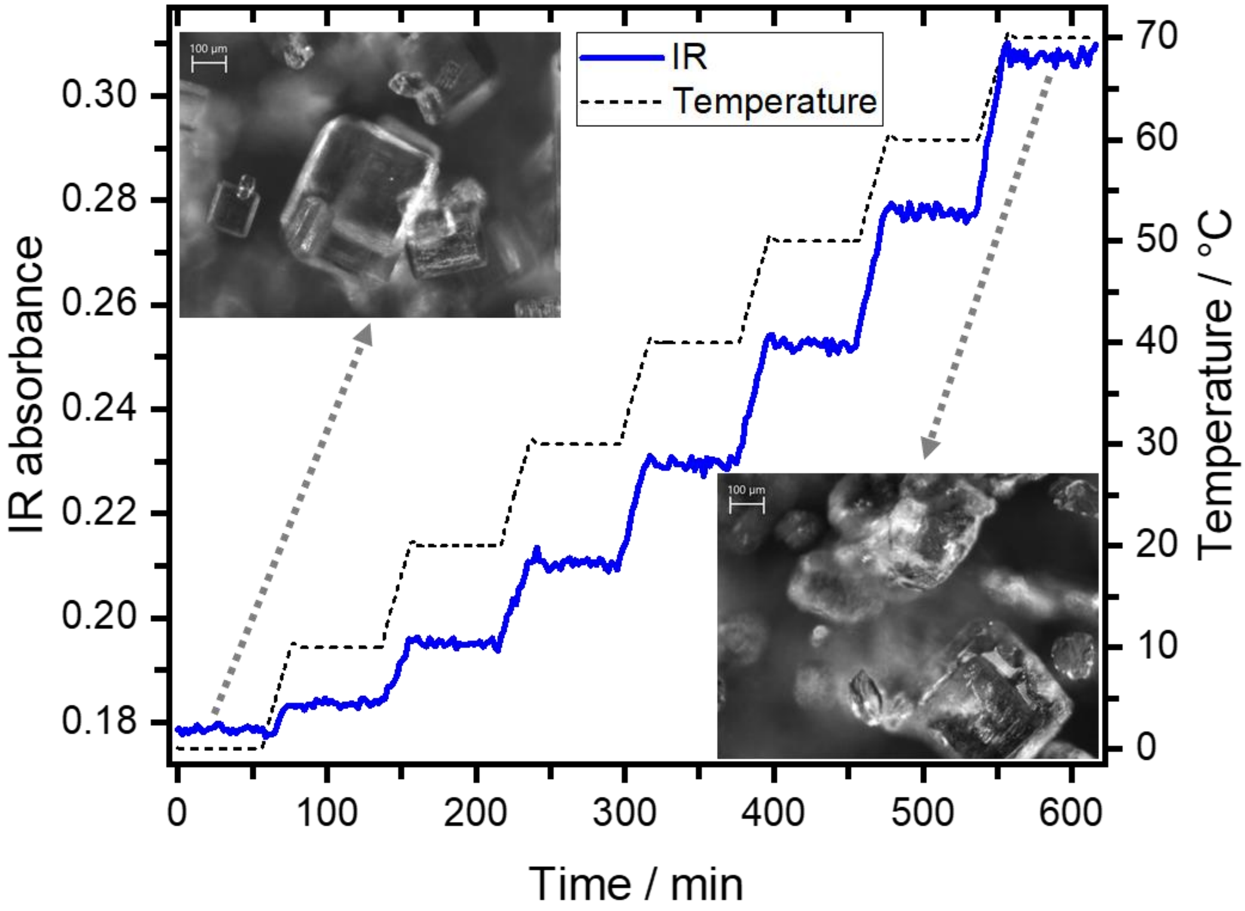

Figure S3. Change in IR absorbance and temperature with time for the variable temperature experiment of a slurry of LAA in MeCN/H_2O (80:20 w/w) for determination of solubility by IR. The trend is taken from the baseline corrected IR carbonyl absorbance band at 1732 to 1678 cm^{-1}. PVM images are shown from the beginning and end of the experiment, with scale bars indicating 100 µm.

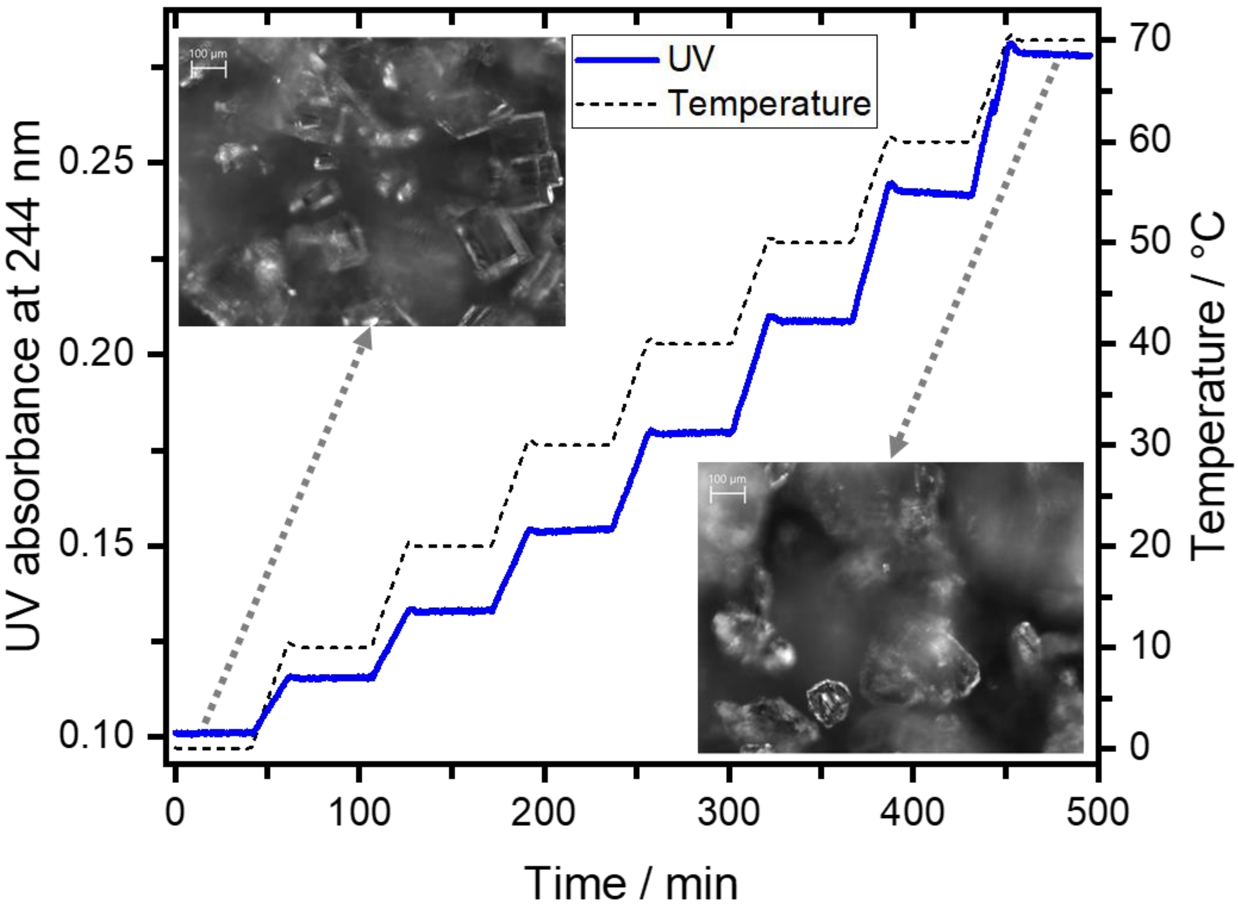

Figure S4. Change in UV absorbance (LAA absorbance at 244 nm) and temperature with time for the variable temperature experiment of a slurry of LAA in MeCN/H_2O (80:20 w/w) for determination of solubility by UV. PVM images are shown from the beginning and end of the experiment, with scale bars indicating 100 µm.

Table S2. Solubility data obtained for LAA in MeCN/H_2O (80:20 w/w) from the gravimetric experiments.

Temperature / °C	Gravimetrically determined solubility / g/100 g MeCN/H_2O (80:20 w/w)			
	Experiment 1	Experiment 2	Experiment 3	Experiment 4
−10.0	4.9154	4.7240	5.0642	4.8866
0.0	5.0964	5.1382	5.1517	5.1740
10.0	5.9373	6.2172	6.4836	6.1964
25.0	8.0230	8.0187	7.9994	7.9931
30.0	8.8181	8.6700	8.7762	8.8792
40.0	10.7128	10.7386	10.7906	10.7198
50.0	13.4236	13.0601	13.2265	13.4715
60.0	16.4553	16.4840	16.6394	16.2492
70.0	21.3374	19.9568	20.2750	20.4888
75.0	23.6971	23.1343	23.3766	22.5230

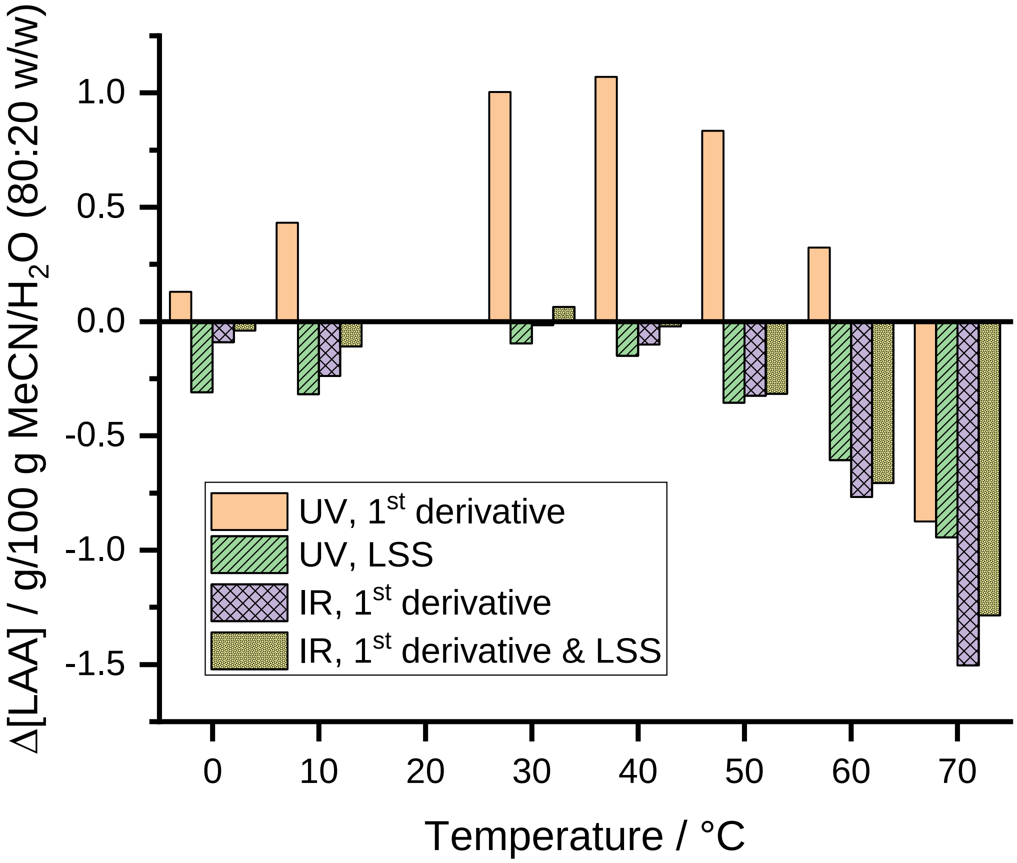

Figure S5 Bias of the solubility determined by UV or IR spectrometry (global models with and without LSS temperature correction) against the solubility determined gravimetrically as a function of temperature.

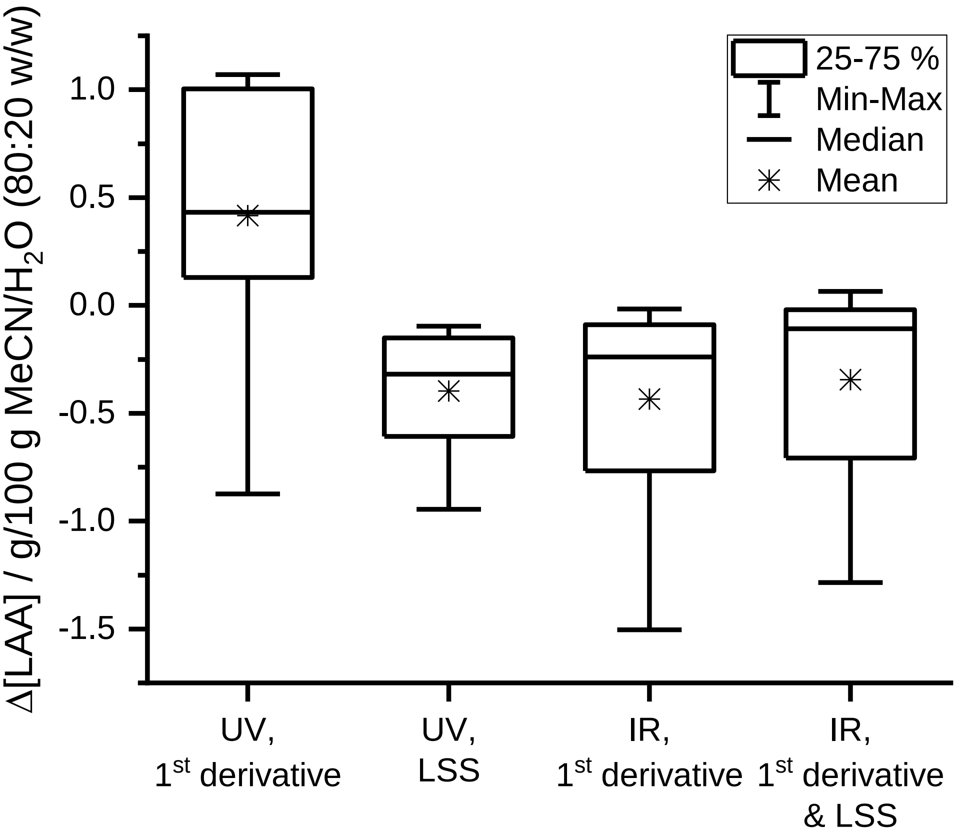

Figure S6 Bias of the solubility determined by UV or IR spectrometry (global models with and without LSS temperature correction) against the solubility determined gravimetrically.
